# Proteomics of Extracellular Vesicles: Recent Updates, Challenges and Limitations

**DOI:** 10.3390/proteomes13010012

**Published:** 2025-03-04

**Authors:** Mohini Singh, Prashant Kumar Tiwari, Vivek Kashyap, Sanjay Kumar

**Affiliations:** 1 Department of Life Sciences, Sharda School of Basic Sciences and Research, Sharda University, Greater Noida UP-201310, India; 2 Division of Cancer Immunology and Microbiology, Medicine and Oncology Integrated Service Unit, School of Medicine, University of Texas Rio Grande Valley, McAllen, TX 78504, USA; 3 South Texas Center of Excellence in Cancer Research, School of Medicine, University of Texas Rio Grande Valley, McAllen, TX 78504, USA; 4 Division of Nephrology, Mayo Clinic, Rochester, MN 55905, USA

**Keywords:** extracellular vesicles, biogenesis, proteomics, EV heterogeneity, cancer, neurodegenerative disorder

## Abstract

Extracellular vesicles (EVs) are lipid-bound vesicles secreted by cells, including exosomes, microvesicles, and apoptotic bodies. Proteomic analyses of EVs, particularly in relation to cancer, reveal specific biomarkers crucial for diagnosis and therapy. However, isolation techniques such as ultracentrifugation, size-exclusion chromatography, and ultrafiltration face challenges regarding purity, contamination, and yield. Contamination from other proteins complicates downstream processing, leading to difficulties in identifying biomarkers and interpreting results. Future research will focus on refining EV characterization for diagnostic and therapeutic applications, improving proteomics tools for greater accuracy, and exploring the use of EVs in drug delivery and regenerative medicine. In this review, we provide a bird’s eye view of various challenges, starting with EV isolation methods, yield, purity, and limitations in the proteome analysis of EVs for identifying protein targets.

## 1. Introduction

Extracellular vesicles (EVs) are small, lipid-bound vesicles derived from cells and secreted into the surrounding environment [1]. These vesicles contain lipids, nucleic acids, and cytosolic and plasma membrane proteins. Based on their biogenesis, size, content, function, and release mechanisms, EVs are classified into three subtypes: exosomes, microvesicles, and apoptotic bodies [2]. EVs play crucial roles in intercellular communication, immune system modulation, cancer progression, and drug delivery [3,4,5]. Exosomes, which are formed by the inward budding of the endosomal membrane, are approximately 50–150 nm in size. Microvesicles (100–1000 nm) and apoptotic bodies (1–5 µm) are generated through the outward budding of the plasma membrane and disassembly of apoptotic cells, respectively [6]. EVs are present in all body fluids, enabling easy sample collection and correlation with disease progression. They have been shown to play critical roles in stem cell expansion, inflammation, and diseases such as neurological disorders, renal disease and various cancer [7,8,9]. When cells take up EVs, specifically exosomes, the cargo is released into the recipient cells, activating downstream signaling pathways. EVs are vital mediators of cell-to-cell communication, transferring diverse bioactive molecules, including proteins, lipids, and nucleic acids, to recipient cells. Among these, proteins—both internal and surface-associated—are pivotal in determining the biological functions of EVs [10]. Surface proteins facilitate EV targeting and uptake by specific cells through receptor–ligand interactions, influencing the specificity of communication pathways [11]. Internal proteins, in contrast, directly modulate cellular processes by delivering functional enzymes, signaling molecules, or transcription factors to recipient cells [12]. Focusing on the proteomic profiles of EVs provides key insights into their roles in both physiological and pathological contexts. For instance, tumor-derived EVs often carry surface integrins that promote metastasis, while immune cell-derived EVs may display MHC molecules that regulate immune responses [13,14]. Furthermore, EV-associated proteins serve as valuable biomarkers for diseases, offering non-invasive diagnostic and prognostic tools [15]. Compared to analyzing the entire EV cargo, targeting the protein components reduces complexity and facilitates a clearer understanding of the functional mechanisms underlying EV-mediated communication. This focus is also crucial for therapeutic advancements, including the engineering of EVs for targeted drug delivery. Thus, the study of EV-associated proteins is indispensable for unraveling their roles in health and disease.

### 1.1. EVs and Their Proteins

**Figure 1:**
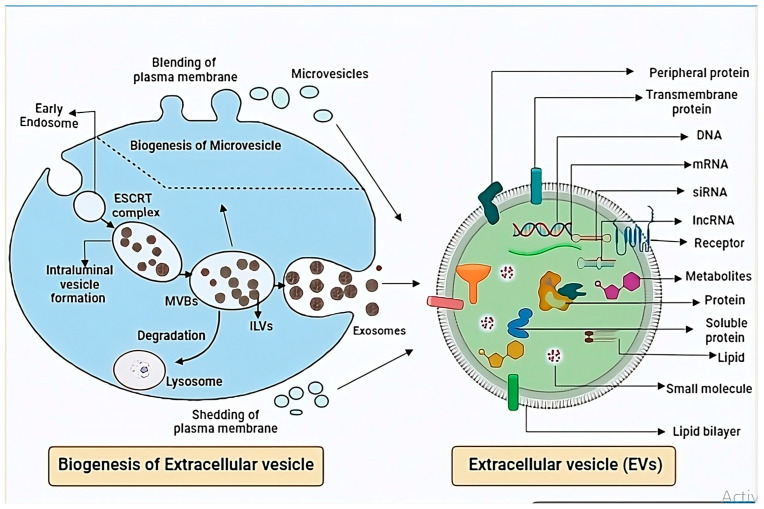
Exosome biogenesis involves multiple stages. Exosomes originate as small vesicles within larger structures known as multivesicular bodies (MVBs). This formation typically involves the ESCRT complex. MVBs then migrate either to the plasma membrane or to lysosomes. At the plasma membrane, MVBs fuse and release exosomes with the assistance of the SNARE complex. In contrast, microvesicles form by directly budding out from the plasma membrane. Apoptotic bodies, produced only by dying cells, break off from the cell surface. These extracellular vesicles are characterized by a lipid bilayer and contain various components such as DNA, RNA, proteins, receptors, lipids, and metabolites.

Molecules present on the surface of EVs play a crucial role in targeting and uptake by recipient cells. These molecules, including proteins, lipids, and glycans, are acquired from parent cells and/or through ionic interactions with extracellular molecules [16] (Figure 1). Surface molecules consist of proteins, lipids, nucleic acids, and glycans, which are either peripherally associated with or integrated into the EV membrane. Membrane proteins contribute to EVs’ stability by protecting them from immune responses [15]. The EV surface proteome comprises a diverse array of proteins with various functions (Table 1). Peripheral proteins associate with the phospholipid head groups through ionic interactions or bind to integral membrane proteins via non-covalent interactions. In contrast, integral membrane proteins span the EV membrane using alpha-helical transmembrane domains [17]. EV proteins include tetraspanins, antigen-presenting proteins, cell adhesion proteins, receptors involved in signal transduction, nucleic-acid-binding proteins, heparin-binding proteins, transport proteins, integrins, and various enzymes (Figure 1).

Membrane proteins perform specific functions based on their origins and targets. For instance, tetraspanins (CD151, CD82, CD81, CD63, tetraspanins 6, tetraspanins 8, and CD9) play critical roles in the ESCRT-independent biogenesis of exosomes, cargo selection, cell targeting, and uptake in both healthy and diseased states [18]. Studies have shown that follicular fluid in humans and the perivitelline space of mouse oocytes contain EVs expressing CD81 and CD9, respectively. The presence of EVs in the reproductive system has been linked to the early developmental stages of follicles and oocytes as well as their subsequent maturation [19,20]. Davidson et al. observed that during hyperglycemic conditions, EVs in human plasma exhibit elevated levels of HSP70 proteins on their surfaces [21]. Similarly, HSP70 and HSP90 proteins were identified on EVs derived from mouse lung carcinoma, contributing to immunomodulation and the release of inflammatory cytokines, which leads to muscle atrophy [22]. VEGF, present on the surface of human umbilical cord mesenchymal-stem-cell-derived EVs, protects against hydrogen-peroxide-induced cell death and lung injury in mice [23]. Conversely, amniotic fluid stem-cell-derived EVs protect against apoptosis by expressing VEGFR1, which binds excess VEGF molecules [24]. As previously discussed, EVs play an essential role in immunomodulation. For example, stem-cell-derived EVs modulate the inflammatory response by suppressing M1 macrophages and promoting the differentiation of M2 macrophages. Additionally, T cell suppression is achieved via AMP-to-adenosine conversion by CD73 proteins present on the surface of stem-cell-derived EVs [25]. Dendritic-cell-derived EVs with MHC complexes on their membranes interact with various immune cells, further demonstrating EVs’ immunomodulatory functions. In cancer, angiogenesis is a critical process that facilitates the supply of oxygen, nutrients, and growth factors to developing cancer cells. EVs regulate the release of proangiogenic factors; for instance, matrix metalloproteases MMP-2 and MMP-9 are activated by the urokinase-type plasminogen activator receptor present on EVs [26]. There are other proteins that are found inside EVs, including cytoskeletal proteins, growth factors and cytokines, heat shock proteins, and proteins involved in MVB biogenesis. Cytoskeletal proteins such as tubulin, cofilin, and actin are involved in exosome biogenesis and secretion, whereas TNF-α, TGF-β, and TRAIL are involved in signaling and the uptake and targeting of exosomes [27].

Several studies have utilized mass spectrometry to analyze proteins present on EVs derived from different cell lines or tissues (Table 1) [17,28,29,30,31,32,33]. Xu et al. isolated exosomes from SW480 colorectal cancer cell lines and identified RNA-binding proteins, RNA nucleoproteins, and membrane proteins [17]. Similarly, Huttman et al. [33] analyzed EV proteins from breast cancer (BC) and non-cancerous cell lines using proteomic profiling and surface protein enrichment. They identified potential biomarkers, such as ST14, CLDN3, and ITGA7, as well as surface-labeled candidates like GSTP1, ADAM10, and RAC2, which were absent in non-cancerous cells. These findings underscore how EVs are a valuable resource for non-invasive biomarker discovery in BC [33]. A study investigated the formation of a protein corona around EVs in blood plasma. EVs isolated from cells and platelets were incubated in plasma and analyzed using various methods. This study revealed higher protein density and newly associated proteins in plasma-coated EVs. Nine shared corona proteins were identified, overlapping with those found on viruses and nanoparticles. Additionally, large protein aggregates were found associated with EV surfaces. Unlike protein aggregates, plasma-protein-coated EVs activated immune responses in dendritic cells, offering new insights into EV research and plasma protein interactions [31]. In certain cases, exosomes or EVs contain proteoforms, adding complexity to intercellular communication. Proteoforms, which are distinct molecular forms of a protein derived from a single gene, arise due to alternative splicing, post-translational modifications, or proteolytic cleavage. For example, Geis-Asteggiante et al. identified proteins in exosomes derived from murine myeloid-derived suppressor cells and found 3 unique S100 family proteins and 44 proteoforms of S100 family proteins [28].

**Table 1:** EV proteins identified through mass-spectrometry analysis.

EV Source	Proteins	Localization	Reference
Seminal fluid	SEMG1, SEMG2, ANPEP, FN1, MME, FASN, CKB, CLU, KRT1, ACTB, TGM4, ALB, ACPP, DPP4, KRT10, PIP, HSP90AA1, HSP90AB1, LTF, MGAM, HSPA8, HSPA1B, KRT2, MYH9, PDCD6IP, KRT9, BASP1, LCP1, KLK3, UBC, DOP1B, YWHAE, TUBB4B, PKM, CNDP2, DYNC1H1, GAPDH, PLPP1, EZR, EEF1A1, RAB3D, RAB27B, HSPB1, HP, ACTN4, ALDOA, TUBB, PGK1, KRT6A, TUBA1C, TACC2, HSPA2, VCP	Surface proteins	[29]
Human primary-monocyte-derived DCs	PPP1R18, TYK2, ITCH, TNIP1, ADD1, CAB39, DAAM1, PIP5K1C, SCRIB, TXNIP, LILRB2, SLC9A3R1, OSTF1, AKAP5, STK24, TSG101, F5 100K, C4A, COL6A3, F2, SERPINF1, F5 10K, ELMO2, PHKB, ABCD1, ABCF1, DHX15, SRP68, TECR, TMEM33, S100A4	-	[30]
Human colorectal cancer cell line SW480	LGALS3BP, RACK1, ARCN1, CAPNS1, CDK5, CLU, DCXR, HNRNPU, DNM1L, EIF3L, EPRS, FKBP1A, GANAB, XPO7, SEC23B, USO1, AIMP2, DDX17, DDX6, DHX9, EIF2S1, EIF3A, EIF3B, EIF3L, EIF4G2, HNRNPC, RPL6, RPL7, RPL9, SNRPD1, TSN, XRCC6	Surface proteins	[17]
Human THP1 cell	HSP90AB1, ITGAL, ACTB, AHSG, ALB, ALDOA, ANPEP, ANXA2, ANXA4, ANXA5, ANXA6, CA2, CAP1, ATP1A1, BASP1, BSG, GNAI2, CD44, CD70, CFL1, CORO1A, CTSG, EZR, FCER1G, GAPDH, GC, GNAI3, GNB1, GNB2, GNB4, GSN, MSN, MYL6, ENO1, HLA-A, HLA-C, HSPA8, HSP90AA1, ITGB1, ITGB2, LDHA, LDHB, LGALS1, LYN, MYH9, MYO1G, NCAM2, PECAM1, PFN1, PGK1, PKM, PPIA, PRDX1, PTPRC/CD45, RAP1A, RAP1B, SLC3A2	Surface proteins	[31]
Human mast cell line HMC-1	AHCYL1, DHRS2, GAPDH, GFPT1, KIF14, NCL, PGAM1, PPP1CB, PRKCB, RACGAP1, RPL8, SERBP1, STUB1, YWHAQ	Peripheral proteins	[32]
MDA-MB-231 and MCF7 breast cancer cell lines	GSTP1, EEF2, DDX10, PGR, RAC2, ADAM10, ACO2, UTP20, N4BP2	Surface proteins	[33]

### 1.2. Biogenesis of EVs

EVs refer to various types of vesicles released into the extracellular environment during normal physiological processes and pathological conditions. The biogenesis of EVs is a highly regulated process involving multiple pathways [34]. Exosomes, a subtype of EVs, are formed through the endosomal system, which consists of early, late, and recycling endosomes. Early endosomes are generated by the invagination of the plasma membrane, which then mature into late endosomes. The inward budding of the late endosomal membrane leads to the formation of intraluminal vesicles (ILVs), which encapsulate cytosolic lipids, proteins, and nucleic acids. Endosomes containing ILVs are referred to as multivesicular bodies (MVBs). MVBs are transported to the plasma membrane, where the ILVs are released into the extracellular environment as exosomes [35] (Figure 1). Exosome biogenesis occurs through either the ESCRT-dependent or ESCRT-independent pathways. The ESCRT-dependent pathway involves five core ESCRT complexes: ESCRT-0, I, II, III, and Vsp4, which facilitate membrane remodeling and vesicle budding. In contrast, the ESCRT-independent pathway relies on other proteins for exosome formation. For example, tetraspanin-enriched microdomains containing CD81, lipid raft domains, flotillin-2, and ARF-6 are critical components of the ESCRT-independent pathway [36].

Microvesicles, also known as ectosomes, are formed by the outward budding (evagination) of the plasma membrane and are independent of the endosomal trafficking pathway. During their biogenesis, rearrangement of the cytoskeletal components and the plasma membrane occurs (Figure 1). Small GTPases, such as ARF1 and ARF6, play key roles in regulating the assembly of cargo and the secretion of cargo into microvesicles [37]. Before membrane pinching, cytoskeletal loosening takes place, along with the sorting of RNA and cytosolic proteins into microvesicles. ESCRT-III, in conjunction with calcium-ion-dependent membrane reorganization and cytoskeleton disassembly, facilitates the pinching-off and release of microvesicles from the plasma membrane [38,39].

Apoptotic bodies constitute another type of EV. They are derived from cells undergoing the final stages of apoptosis. Their formation involves tightly regulated morphological changes (Figure 1). These vesicles contain glyco-epitopes, histones, and DNA fragments that contribute to immunological tolerance and anti-inflammatory responses [40].

## 2. Proteomics of EVs

In recent years, various tools and techniques have been explored to study the proteins involved in signaling pathways that regulate healthy and diseased states of the cell. Among these, proteomic analysis has emerged as a powerful approach for studying and identifying protein targets. Proteomic analysis of cancer tissues using mass spectrometry has led to the discovery of numerous cancer-specific biomarkers that aid in the detection and treatment of both early- and late-stage cancers [41]. Obtaining tissue samples from cancer patients for proteomic analysis is an invasive process, whereas obtaining fluid samples—such as plasma, urine, or ascitic fluid—is a non-invasive alternative for identifying cancer biomarkers. EVs, nanosized particles found in body fluids, are directly associated with tumor progression. For example, the oncogenic microRNA miR-222 is overexpressed in melanoma cells and, when transferred to primary melanoma cells via EVs, activates the PI3K/Akt pathway and downregulates p27, promoting cell proliferation and cancer progression [42]. Similarly, EVs derived from malignant hepatocellular carcinoma (HCC) cells carry mRNAs and oncogenic proteins. When taken up by HCC cells, these EVs activate the MAPK and PI3K/AKT pathways, enhancing hepatocyte invasion and migration by increasing the secretion of active MMPs [15]. The analysis of exosomes from various body fluids has identified numerous proteins, including the Rab protein family, annexins, integrins, CD63, LAMP-1/2, HLA class I and II molecules, enzymes, heat shock proteins, and drug transporters [43]. The protein composition of exosomes varies depending on the physiological or pathological state of the cells from which they are derived. For instance, exosomes obtained from T cells are characterized by the presence of CD3. The methods used for EV isolation are critical to proteomic analysis, as the isolation of EVs from cell culture media or body fluids is hindered by potential protein contaminants. Over the years, methods for isolating EVs have been continuously refined. However, no existing protocol can recover a completely pure EV subpopulation. Most current protocols significantly influence the downstream omics results [27]. Several studies have examined different methods for EV isolation, each with its own advantages and limitations. For instance, Brennan et al. [44] evaluated various techniques for isolating EVs from human serum to identify methods that can effectively separate EVs from protein and lipid contaminants. Their comparison of techniques—including ultracentrifugation, size exclusion chromatography (SEC), polymer-based precipitation, and density gradient centrifugation—revealed that while methods such as SEC and ExoQuick yield high EV counts, they often include contaminants like lipoproteins. Combining methods, such as SEC with ultracentrifugation, improves purity but reduces yield, underscoring the need to balance EV purity and yield depending on the intended downstream applications. Similarly, a study by Veerman et al. [45] evaluated EV isolation methods across two sample types: conditioned media from MM6 cells and human plasma. Their findings highlight the significant impact of isolation methods on EV yield, purity, and subpopulation diversity. For example, ExoQuick is effective for small plasma volumes but less effective for conditioned media, whereas Izon 35 isolates more EV proteins but introduces significant non-EV contamination.

### 2.1. Various Methods for Isolation of EVs

In recent years, research has increasingly focused on extracellular vesicles (EVs) as potential prognostic and diagnostic markers as well as vehicles for drug delivery. A significant challenge faced by researchers is determining an optimal, reliable, and efficient method for isolating EVs. Commonly used isolation techniques include centrifugation, filtration, and affinity-based methods (Table 2). Achieving a pure population of EVs often requires the combination of multiple isolation techniques to maximize both purity and yield.

#### 2.1.1. Ultracentrifugation

Ultracentrifugation is a widely used method for isolating particles based on their size, shape, and density (Table 1). This process separates various components within a sample by applying centrifugal force, which facilitates the isolation of individual components. Ultracentrifugation plays a crucial role in isolating exosomes, microvesicles, and apoptotic bodies. During EV isolation, differential centrifugation is employed, involving successive rounds of centrifugation at increasing speeds to progressively remove larger debris and isolate smaller EVs. Initially, large cells and cell debris are removed at low speeds (300–500 g for 10 min). Subsequently, cell organelles and other large particles are separated at 2000 g for 10–20 min. Microvesicles and other larger particles are removed via centrifugation at 10,000–20,000× *g* for approximately 30 min. Finally, exosome isolation is achieved through ultracentrifugation at 100,000–120,000× *g* for 1–2 h. Despite its widespread use, ultracentrifugation has certain limitations. It is time-consuming, requires extensive handling, and may result in co-precipitation of lipoproteins with a sample. Additionally, the high centrifugal forces involved can compromise the integrity of the EVs [46]. To overcome these challenges and improve the purity of the isolated EVs, density gradient centrifugation has been developed as an advanced refinement of the technique.

In density gradient ultracentrifugation, molecules or particles are separated based on their buoyant density. This technique involves layering a biocompatible medium, such as iodixanol, with varying densities, from top to bottom in a centrifuge tube. The sample is placed on top of the gradient and then subjected to centrifugation at 100,000× *g* for approximately 16 h [47]. This method enhances the purity of the EVs by isolating them with minimal contamination, but it has its own limitations. It is time-consuming and requires large quantities of biological samples. Additionally, this technique may impact the subpopulation of EVs, leading to the loss of large vesicle populations. Neilson et al. successfully used differential ultracentrifugation to isolate EVs with procoagulant activity from blood, with minimal contamination from lipoproteins and plasma proteins [48]. It was shown that iodixanol is preferred over sucrose, as EVs reach equilibrium more quickly in the former medium than in the latter [49].

#### 2.1.2. Size Exclusion Chromatography

This method separates molecules or particles based on their hydrodynamic radius. It involves the use of a column with a stationary phase, onto which a liquid mobile phase containing the sample is allowed to pass. The sample exits the column at a rate proportional to its particle size. Larger particles pass through the column via voids, while smaller particles are retained within the gel pores and subsequently eluted with the mobile phase [50]. Size exclusion chromatography (SEC) results in higher recoveries and greater purity when combined with other isolation techniques, such as differential centrifugation and ultrafiltration [51,52]. The combination of ultracentrifugation and size exclusion chromatography significantly enhances the proteomic profiling of exosomes isolated from plasma while also reducing contaminant levels [8].

#### 2.1.3. Ultrafiltration

Ultrafiltration is a process in which membranes with varying molecular weight cutoffs (MWCO) are used to separate molecules or particles based on their sizes. Unlike ultracentrifugation, ultrafiltration does not require specialized equipment, so it results in shorter processing times and higher output. However, this method has limitations, such as the potential for membrane breakage and the loss of EVs due to deformation caused by mechanical shear forces. To overcome these limitations, two methods have been developed based on ultrafiltration principles: tangential flow filtration and sequential filtration. In sequential filtration, a sample first passes through a 1000 nm filter, followed by a 500 kDa MWCO, and then a 200 nm filter to remove cellular debris, free proteins, and isolate exosomes, respectively [53]. In tangential flow filtration, a sample is applied in a direction parallel to the membrane, minimizing damage while maintaining the integrity and purity of the EVs. Both methods are scalable, less time-consuming, and cost-effective, making them ideal for large-scale EV isolation. Additionally, they help prevent membrane damage and preserve EVs during the process. Chernyshev et al. devised an asymmetric depth filtration technique for EV isolation, where larger particles are retained at the top of the medium, while smaller particles—such as EVs—are isolated from the porous medium below [54].

#### 2.1.4. Polymer-Based Precipitation

This method involves mixing a polymer solution with a sample, which results in the formation of a polymer network at an optimum temperature and salt concentration. This polymer network aids in the isolation of EVs through centrifugation at low speeds. Several commercial kits are available for this purpose, including the Total Exosome Isolation Kit from Thermo Fisher Scientific, Waltham, Massacheusetts and ExoQuick™ from System Biosciences, Palo Alto, California. After centrifugation, PBS is used to resuspend the isolated EVs for subsequent analyses. One drawback of this method is that the isolated EVs often have a high salt concentration, which can interfere with proteomic analysis using mass spectrometry. To overcome this, an additional step involving 1D gel electrophoresis and membrane filtration can be used to clean the sample. A study conducted by Niu et al. demonstrated that EVs isolated from endometrial cell lines using polymer-based precipitation techniques were both pure and biochemically active [55]. While this method increases the yield of EVs, it results in lower purity. To obtain a more purified sample, downstream processing using size exclusion chromatography is often performed.

#### 2.1.5. Immunoaffinity-Based Isolation

Immunoaffinity is based on the principle of antigen–antibody interaction. The surface proteins present on the EVs act as antigens, while corresponding antibodies are attached to magnetic beads. Upon incubation with the sample, an antigen–antibody complex is formed, which can be separated using magnetic fields [56]. This technique is highly specific and sensitive, resulting in a higher yield of EVs. For instance, compared to ultracentrifugation, the isolation of EVs from plasma samples was enhanced 15-fold using immunoaffinity [57]. However, a limitation of this method is the requirement for specific protein markers on the EV population and the availability of suitable antibodies [58].

#### 2.1.6. Microfluidics

Microfluidic technology processes small volumes of fluidic samples, integrating the isolation, detection, and analysis of EVs. Micro- and nano-fabricated channels are used for isolation. One can utilize an immune affinity technique, where antibodies are immobilized on the surface of the microfluidics, or rely on technologies like magnetic waves, acoustic waves, and dielectric electrophoresis [59]. For example, a microfluidic device using CD63 antibodies and polydimethylsiloxane, known as ExoChip, has been developed [60]. The advantages of this technique include its high specificity, sensitivity, rapid processing, purity, and compatibility with low sample volumes. However, the limitations include potential clogging and the inability to process large sample volumes efficiently.

**Table 2:** Different isolation techniques with their pros and cons.

Isolation Technique	Principles	Time	Disadvantages	Advantages	Reference
Differential centrifugation	Density and size	250 min–2 days	Expensive setup, may lead to disintegration of the EVs, heterogenous population	Can be used for large and small samples, equipment is easy to operate	[61]
Size exclusion chromatography	Size	Column washing and 1 mL/min	Cracking of the medium, EV deformation	No requirement for special equipment, purity is better than centrifugation method, reduced sample-processing time	[62]
Polymer precipitation	Polymer hydrophilicity	65 min	Low purity, contamination by the polymer and salts	Simple, rapid, does not require special handling	[63]
Ultrafiltration	Size	130 min	Clogging and deformation of the EVs due to mechanical shearing	High yield and purity, cost- and time-effective, reproducible	[64]
Immunoaffinity	Antigen–antibody interaction	240 min	Antibodies are expensive	High purity	[65]
Microfluidics	Electrical, viscoelastic, acoustic	5–40 min	Fast, precise, simple, and cost-effective	Clogging	[66]

## 3. Various Challenges

EVs are small particles that are secreted by cells, and EV proteomics entails the analysis of the proteins present within the vesicles. Accurate, reproducible, and reliable protein quantification techniques are crucial for EV-based protein research. However, proteomic investigations are affected by challenges faced during the isolation, purification, and downstream processing of the EVs. Based on different studies, it has been concluded that EVs are heterogeneous in size, origin, and composition. Differences in size affect the single-step isolation of the majority of EVs, such as when using size exclusion chromatography with sepharose 7B; EVs with a size range of 70 nm and above are retained, and the smaller EVs are eluted with soluble proteins and lipoproteins [67]. It is quite challenging to differentiate between different EVs during isolation and characterization. EV heterogeneity further affects the reproducibility and interpretation of the proteomic results. For instance, isolation of specific subpopulations with specific characteristics is difficult, resulting in mixed samples that can negatively affect downstream processing.

The availability of various methods for EV isolation leads to differences in the yield and purity of EVs under different laboratory conditions. This variability ultimately affects the quality and comparability of proteomics data obtained from the same sample across different laboratory setups. A study conducted by Cringa et al. showed a difference in the yield of exosomes due to interlaboratory differences. It was indicated that even though the samples and the methods were the same, there is a need for experimental parameter standardization, such as k-factor and speed [68]. Another study conducted by Tiruvayipati et al. indicated a difference in the protein content between biological and technical replicates from lung adenocarcinoma cells. In the case of these replicates, it was observed that certain proteins were present in one replicate and absent in another replicate [69]. Exosomes, derived from MVBs, contain certain proteins in low abundance, and it is quite difficult to analyze these proteins at low levels through mass spectrometry. In some cases, during isolation, proteins from plasma, cells, or other fluid samples can co-isolate with EVs acting as a contaminant. Uromodulin, casein, albumin, and lipoproteins are common contaminants derived from urine-, milk-, plasma-, and blood-derived EVs [70,71]. These contaminating proteins may interfere with the proteomic analysis of the EV samples, resulting in inaccurate EV protein data. Further, this contamination also affects the identification of biomarkers and the delineation of the functions of these proteins in signaling pathways or cell–cell communication [72]. In the case of neurodegenerative disorders, certain biomarkers help in determining disease states; for example, PrP, tau, and cystatin are found in the exosomes derived from the CSF of patients with prion disease, and SOD1, FUS, and TDP-43 are found in the plasma, CSF, and serum of patients with amyotrophic lateral sclerosis [73,74,75,76]. Besides the soluble proteins and lipoproteins, the chemicals or molecules used for precipitation or density gradients may act as contaminants during mass-spectrometry analysis [77]. In the human genome, approximately 10–20 proteoforms, or protein species, are produced from 20,000 genes, enhancing the proteome’s complexity. Thus, the human proteome can have around 1 million proteoforms. Proteoforms are less abundant on the surfaces of EVs and, in some cases, serve as biomarkers. However, conventional protein detection technologies lack the sensitivity required to detect these low-abundance proteoforms [78]. Since proteoforms represent isoforms of a protein, they do not have unique peptide sequences referred to as proteotypic peptides. During proteomic analysis, the shared peptides are either assigned to a single protein or excluded from the study [79].

Unlike transcriptomics analysis, there is no protein amplification technique, and thus large amounts of EVs are required for analysis, which further requires large sample volumes. Complex and large datasets are generated during proteomic analyses of EVs, and thus advanced computational tools are needed to analyze, identify, and functionally validate the EV proteome. With more research on EV proteomes, various databases including information on EV proteomic profiling are being generated. An EV proteome database was reported by Hoshino et al., with approximately 426 human samples [80].

## 4. Various Solutions for the Challenges

Based on our understanding and the ways in which different EV isolation methods work, in order to avoid heterogeneity in EV populations and enhance purity, combined use of various isolation techniques would be helpful [81]. For instance, ultracentrifugation, followed by polymer precipitation and size exclusion chromatography or immunoaffinity, can be used to derive a specific subpopulation of EVs. Zhang et al., in their study, used three isolation methods sequentially, resulting in the isolation of EVs from plasma with high purity. Their approach included PEG precipitation, iohexol gradient centrifugation, and size exclusion chromatography and led to 71% recovery with no contaminating proteins [82].

Asymmetric-flow field fractionation (AF4) was used to segregate EVs to reduce size heterogeneity. In this approach, exosomes were subjected to a forward laminar channel flow and variable crossflow, leading to separation based on their hydrodynamic properties and densities. The segregated exosomes were further classified based on their sizes: as exo-L (90–120 nm) and exo-R (60–80 nm) [83]. A method was devised by Cross et al. [84] for the isolation of EVs from a very minimal amount of a sample. The developed comparative proteomic pipeline improved several aspects of EV proteome analysis, such as by optimizing sample preparation, reducing chromatography times, and utilizing data-independent acquisition. This innovation enabled a 20–40-fold reduction in the required sample size (<1 µg starting EV protein) and allowed for the completion of sample preparation, quantification, and acquisition within a single day.

Extracellular vesicle (EV) isolation methods significantly influence the outcomes of downstream proteomic analyses by affecting the yield, purity, and integrity of isolated EVs. Techniques such as ultracentrifugation, size exclusion chromatography (SEC), and ultrafiltration are commonly employed but vary in their ability to minimize contamination from non-EV proteins, such as albumin and lipoproteins. For instance, ultracentrifugation, a gold-standard method, often yields EVs with high recovery rates often leads to the co-isolation of protein aggregates, leading to proteome contamination [85]. In contrast, SEC offers improved purity by separating EVs based on size, though it may result in lower yields and partial loss of smaller vesicles [86]. Similarly, ultrafiltration can rapidly concentrate EVs but introduces shear forces that may damage vesicle integrity and alter the proteomic profile [87]. Such variabilities in EV isolation can hinder the identification of true EV-associated biomarkers and lead to misinterpretation of results. Consequently, selecting an appropriate isolation technique tailored to specific research objectives and downstream applications is critical for ensuring accurate proteomic analyses. Future advances in isolation technologies and standardization across protocols are essential to mitigate these challenges and enhance the reliability of EV-based proteomics. For instance, a 2024 study introduced an enzyme-free protocol for isolating brain-derived EVs, demonstrating minimal alterations in proteomic profiles compared to enzyme-assisted methods, which can introduce proteolytic modifications [88]. Furthermore, they developed a streamlined EV-based proteomics strategy for early colorectal cancer diagnosis, emphasizing the importance of efficient and reproducible EV isolation to achieve comprehensive proteome coverage and accurate biomarker identification [88].

In the conventional bottom-up approach to proteomics, proteins are not segregated based on their isoforms; instead, prior to mass spectrometry analysis, the approach provides only limited information about the amino acid sequence. In contrast, in the top-down approach, the proteoforms are segregated by using techniques like 2D gel electrophoresis and gel-eluted liquid fraction entrapment electrophoresis prior to mass spectrometric analysis [89,90]. This gives information about the proteoforms, different protein species present in EVs.

Extracellular vesicle (EV) proteomics has emerged as a vital field for understanding the biological and clinical roles of EVs, and it is driven by advancements in analytical technologies. Quantitative proteomic approaches such as label-free quantitation (LFQ) and labeling methods have been extensively utilized in EV studies. LFQ enables the comparison of peptide signal intensities without the need for chemical modifications, offering simplicity and compatibility with diverse sample types [91]. In contrast, labeling approaches, including isobaric tags for relative and absolute quantification (iTRAQ) and tandem mass tags (TMTs), provide multiplexing capabilities with enhanced accuracy, making them suitable for comparative analyses under multiple conditions [91].

Instrument-specific methodologies have further expanded the capabilities of EV proteomics. Orbitrap-based proteomics offers high resolution, excellent mass accuracy, and a wide dynamic range, facilitating the identification of low-abundance proteins in complex EV samples [92]. Alternatively, MALDI-TOF (Matrix-Assisted Laser Desorption/Ionization Time-of-Flight) mass spectrometry excels in rapid protein identification and high-throughput profiling, making it especially advantageous for large-scale EV studies [93]. A novel method for profiling single EV proteins using squeezable methacrylated hyaluronic acid hydrogel microparticles (MHPs) was introduced. These MHPs serve as scaffolds for immobilizing EVs, enabling a highly sensitive, multiplexed analysis using integrated rolling circle amplification (RCA). By compressing the MHPs, amplified signals are aligned for easy visualization, offering a scalable and high-throughput approach to studying EV heterogeneity. The method is validated by profiling cancer cell markers, demonstrating its potential for advancing molecular diagnostics [94]. These diverse technologies provide complementary strengths, enabling comprehensive and robust proteomic analyses of EVs and advancing the understanding of their molecular complexity.

In recent years, EV proteomics has progressed, with respect to enhancements in the exosome isolation method and the development of advanced instrumentation for proteomics analysis and enhanced sensitivity for detection. But there are still many aspects in EV proteomics that need attention. So far, there is no standard protocol for EV characterization from in vivo and in vitro environments with similar functions. Furthermore, these technical challenges need to be dealt with in order to use EVs as diagnostic and prognostic markers and design novel cancer therapies.

## 5. Future Perspectives Regarding Exosomes in Medical Sciences

While the characterization of EVs based on their physical and biochemical properties is important, the functional analysis of EVs will help to assess their roles in biological systems. The main problems affecting the biological applications of EVs include yield, purity, and their integrity. Any changes in an EV’s structure will affect its analysis, and thus it is very crucial to optimize isolation techniques to understand EVs’ functions and effects on various cell processes. When one is comparing healthy and diseased states, EVs can serve as diagnostic and prognostic tools. Besides this, they can be used for drug delivery, where therapeutic drugs can reach the target cells [95]. This will prevent the degradation of the drugs and result in targeted delivery, thereby enhancing a drug’s efficiency and decreasing the chances of side effects. In the field of regenerative medicine, EVs isolated from stem cells can be used for the treatment of various ailments, including neurological disorders, sepsis, and cardiovascular and kidney diseases [96,97]. For instance, mesenchymal stem cell (MSC)-derived EVs have been shown to ameliorate cardiac injury by reducing inflammation and fibrosis while promoting neovascularization [98].

It has been demonstrated in various studies that EVs are important for intercellular communication between cancerous and non-cancerous cells [33]. EVs in the plasma of cancer patients can phenotypically affect the immune cells in the cancer microenvironment. For instance, EVs derived from breast cancer cells change the phenotype of T cells to Treg cells through TGF- β mediated phosphorylation of STAT3 and Smad2/3 [99]. EVs originating from ovarian, pancreatic, and colon cancers reprogram cancer-suppressive M1 macrophages into tumor-supportive M2 macrophages [100,101]. Thus, it is important to assess the immunomodulatory roles of EVs and their proteins in cancer progression. One can also study the effects of EVs on immune cells in other diseases such as Parkinson’s disease. Characterization of EV subpopulations and their biochemical markers is very important. Rapid and accurate protein identification and quantification methods need to be used in the EV research field. Improvement is required in determining EVs’ proteome, their mutations, and post-translational modifications in order to understand their biological functions. Metabolomics, transcriptomics, and lipidomics, along with proteomics, will help in better understanding EV systems’ biology. In the future, EV protein identification in consideration of the pathophysiological states of individuals will help aid translational therapy.

## Data Availability

Not applicable.
